# Supplementary material for: MetaRibo-Seq measures translation in microbiomes
Source: Nat Commun. 2020 Jun 29;11:3268. doi: 10.1038/s41467-020-17081-z (PMC7324362; doi:10.1038/s41467-020-17081-z)
Supplement: Supplementary file 10 — Supplementary Data 7 [file 41467_2020_17081_MOESM10_ESM.zip › File2/Confidence_High_Taxonomy/150526_out.krona.html]

Javascript must be enabled to view this page.

members
magnitude
magnitudeUnassigned
count
unassigned
taxon
rank

150526\_out

2

superkingdom
2
2

phylum
976
2

200643
2
class

order
2
171549

1
815
family

1
816
genus


SRS143417\_contig\_number\_9586
species
1
2292953

1
1853231
family

genus
1
574697

1
2093856

SRS142890\_contig\_number\_12174
species
